# Association of Relaxin-1 Levels with Mortality in Sepsis and Septic Shock

**DOI:** 10.3390/jcm15124661

**Published:** 2026-06-16

**Authors:** Seyda Kayhan Omeroglu, Ozden Yildirim Akan, Huseyın Ozkarakas, Ferhat Demirci, Ismail Demir

**Affiliations:** 1 Department of Anesthesiology and Reanimation, İzmir Faculty of Medicine, University of Health Sciences, 35540 İzmir, Türkiye; huseyin.ozkarakas@sbu.edu.tr; 2 Department of Internal Medicine, İzmir Faculty of Medicine, University of Health Sciences, 35540 İzmir, Türkiye; ozden.yildirimakan@sbu.edu.tr (O.Y.A.); drismaildemir22@gmail.com (I.D.); 3 Department of Biochemistry, Tepecik Training and Research Hospital, 35020 İzmir, Türkiye; drdemirci05@gmail.com

**Keywords:** relaxin-1, septic shock, biomarkers, endothelial dysfunction, prognosis

## Abstract

**Background/Objectives**: Hemodynamic disturbances in sepsis and septic shock arise from the vasoactive effects of inflammatory mediators involved in the immune response. Relaxin-1 is a pleiotropic hormone associated with inflammation, angiogenesis, tissue repair, and vasodilation. This study aimed to investigate the changes in relaxin-1 levels in septic shock and to evaluate their association with mortality. **Methods**: This prospective observational study was conducted in a Level II intensive care unit. Demographic characteristics, vital signs, APACHE II and SOFA scores, comorbidities, and routine laboratory parameters were recorded at admission and at 48 h. Serum relaxin-1 levels were measured at both time points and analyzed in relation to survival status. Binary logistic regression was additionally performed to evaluate variables associated with mortality in a multivariable framework. **Results**: A total of 48 patients with sepsis and septic shock were included (54.2% female; mean age 73.4 ± 14.7 years). Overall mortality was 33.3%. Relaxin-1 levels significantly increased from baseline (11.25 ± 4.85 pg/mL) to 48 h (12.64 ± 4.81 pg/mL) (*p* = 0.047). Baseline relaxin-1 levels were significantly higher in non-survivors compared to survivors (14.62 ± 4.47 pg/mL vs. 11.65 ± 4.73 pg/mL, *p* = 0.043). **Conclusions**: Elevated Relaxin-1 levels were associated with mortality in patients with sepsis and septic shock. The observed increase in Relaxin-1 during early follow-up suggests a potential link with the underlying pathophysiological processes. Although Relaxin-1 was associated with mortality, its independent prognostic value could not be established in multivariable analysis due to the limited sample size. Larger, adequately powered multicenter studies are required to confirm these findings.

## 1. Introduction

Sepsis is a life-threatening condition characterized by organ dysfunction resulting from a dysregulated host response to infection, and its underlying mechanisms are not yet fully understood [1]. In septic shock, profound alterations occur in immune, inflammatory, and endothelial pathways, leading to dysregulation of vascular tone, impaired tissue perfusion, and circulatory failure [2,3,4]. These disturbances are closely associated with multi-organ dysfunction and increased mortality, particularly in critically ill patients with advanced age and comorbidities [5].

Despite advances in critical care, prognostic assessment in septic shock remains challenging. Widely used clinical scoring systems such as SOFA and APACHE II provide valuable information; however, they primarily reflect established organ dysfunction rather than the underlying vascular and endothelial pathophysiology. Therefore, there is an ongoing need for biomarkers that capture these mechanisms and may provide complementary prognostic information.

Relaxin peptides constitute a family of structurally related hormones with diverse biological functions. Among these, relaxin-2 (RLN2) is the primary circulating form in humans and exerts its effects mainly through the relaxin family peptide receptor-1 (RXFP1), which is expressed in vascular and endothelial tissues [6,7]. Activation of the relaxin–RXFP1 pathway promotes vasodilation, enhances nitric oxide production, modulates inflammatory responses, and improves endothelial function [8,9]. In experimental models, relaxin has been shown to increase cardiac output, improve tissue perfusion, and exert protective effects against ischemia–reperfusion injury [10].

These vasodilatory, anti-inflammatory, and endothelial-modulating properties suggest that relaxin may be upregulated in response to hemodynamic stress and systemic inflammation in sepsis, reflecting a compensatory mechanism aimed at preserving vascular function and tissue perfusion. However, commonly used clinical scoring systems and conventional biomarkers such as CRP and procalcitonin primarily reflect inflammatory activity and may not fully capture the interplay between vascular dysfunction and systemic inflammation [11]. Considering the central role of endothelial dysfunction and microcirculatory impairment in septic shock, biomarkers that reflect both hemodynamic and inflammatory pathways are of particular interest. In this context, the relaxin–RXFP1 pathway may represent a biologically relevant target, as it integrates vascular, endothelial, and inflammatory signaling mechanisms.

Given that endothelial dysfunction and microcirculatory impairment are central features of septic shock, the relaxin–RXFP1 pathway represents a biologically plausible link between vascular dysregulation and clinical outcomes. However, the clinical relevance of circulating Relaxin-1 levels in septic shock remains unclear, and data on its prognostic role are limited.

The primary aim of this study was to evaluate the association between baseline circulating Relaxin-1 levels and ICU mortality in patients with sepsis and septic shock. A secondary aim was to assess temporal changes in Relaxin-1 levels during the first 48 h of ICU admission. In addition, we explored the relationship between Relaxin-1 levels and established disease severity indicators.

## 2. Materials and Methods

### 2.1. Study Design and Population

This prospective observational study was conducted in the Level II Intensive Care Unit of Bozyaka Training and Research Hospital. The study protocol was approved by the Institutional Ethics Committee (Approval No: 18; Date: 27 March 2025).

Patients aged 21–95 years who were diagnosed with sepsis or septic shock according to Sepsis-3 criteria and admitted to the intensive care unit were included in the study. In accordance with current sepsis management principles, patients requiring fluid resuscitation and/or vasopressor support were included. Patients with oncological or hematological malignancies, those receiving chemotherapy, radiotherapy, or chronic immunosuppressive therapy, and pregnant women were excluded to avoid potential confounding effects on inflammatory and hormonal responses. In addition, patients in whom corticosteroid therapy was initiated at baseline or during the 48 h follow-up period were excluded in order to minimize potential confounding, as corticosteroids may influence Relaxin-1 levels and inflammatory responses.

Although most patients were clinically considered to have septic shock at ICU admission, six patients (12.5%) did not fulfill strict Sepsis-3 septic shock criteria at the time of retrospective data verification and were therefore classified as having sepsis. This discrepancy likely reflects the dynamic nature of early hemodynamic instability and evolving vasopressor requirements during the initial phase of critical illness and has been acknowledged as a limitation of the study.

The primary outcome of the study was ICU mortality, defined as death occurring during the ICU stay. Accordingly, patients were categorized as survivors or non-survivors based on ICU outcome at the time of discharge or death.

Clinical and laboratory data were evaluated at admission and at 48 h after admission. Demographic characteristics, vital signs, APACHE II and SOFA scores, and comorbidities were recorded for all patients. Routine hematological, biochemical, and inflammatory parameters were measured at both time points. Serum Relaxin-1 levels were also measured at admission and at 48 h.

Treatment-related variables, including vasopressor use, fluid resuscitation, antimicrobial therapy, and corticosteroid administration, were not systematically recorded or included in the analysis. Therefore, their potential confounding effects could not be evaluated.

### 2.2. Relaxin-1 Measurement

Serum Relaxin-1 levels were measured using a commercially available Human Relaxin-1 (RLN). Human Relaxin-1 (RLN) ELISA kit (MyBioSource, San Diego, CA, USA; Catalog No: MBS702180). The assay detection range was 6.25–400 pg/mL, with a detection limit of <1.56 pg/mL. The intra-assay coefficient of variation (CV) was <8%, and the inter-assay CV was <10%. All procedures were performed strictly according to the manufacturer’s instructions, and results were expressed in pg/mL.

Blood samples were collected at ICU admission (baseline) and at 48 h, centrifuged, and stored at −80 °C until batch analysis was performed after completion of patient enrollment. All samples were analyzed in a single measurement without duplicate testing. Laboratory personnel were blinded to patient outcomes.

### 2.3. Statistical Analysis

Statistical analyses were performed using IBM SPSS Statistics version 27 (IBM Corp., New York, NY, USA). The normality of data distribution was assessed using the Kolmogorov–Smirnov and Shapiro–Wilk tests.

Normally distributed variables were analyzed using paired *t*-tests, whereas non-normally distributed variables were analyzed using the Wilcoxon signed-rank test. Differences between survivors and non-survivors were analyzed using the Mann–Whitney U test. Categorical variables were compared using Pearson’s chi-square test.

Receiver operating characteristic (ROC) curve analysis was performed to evaluate the predictive value of Relaxin-1 for mortality. A *p*-value < 0.05 was considered statistically significant.

Given the exploratory nature of the study and the limited sample size, no formal correction for multiple comparisons was applied. Therefore, *p*-values should be interpreted with caution.

To evaluate independent predictors of mortality, binary logistic regression was performed with ICU mortality as the dependent variable. Due to the limited number of outcome events (*n* = 16), the multivariable model was restricted to two predictors to minimize the risk of overfitting and maintain an adequate events-per-variable ratio.

APACHE II score and serum albumin were selected as covariates based on their clinical relevance and univariate significance. Results are reported as odds ratios (ORs) with 95% confidence intervals (95% CIs).

The discriminative ability of APACHE II was additionally assessed by ROC curve analysis for comparison with Relaxin-1. All regression analyses should be considered exploratory and hypothesis-generating.

All analyses were performed in the overall cohort, and no subgroup analysis between sepsis and septic shock patients was conducted due to the limited sample size.

### 2.4. Use of Artificial Intelligence Tools

During the preparation of this manuscript, ChatGPT (OpenAI, GPT-5.5) was used exclusively for English language polishing, grammar correction, and improvement of sentence clarity. The authors reviewed and edited all generated content and take full responsibility for the final manuscript. No artificial intelligence tools were used for study design, data collection, statistical analysis, interpretation of results, or generation of scientific conclusions.

## 3. Results

### 3.1. Baseline Characteristics of Patients

A total of 48 patients were included in the study. Of these, 54.2% (*n* = 26) were female and 45.8% (*n* = 22) were male. The mean age was 73.4 ± 14.7 years (range: 21–92), with a median age of 75 years. Half of the patients (50%) were admitted directly to the intensive care unit (ICU), while 31.3% were referred from hospital wards and 18.8% were transferred from other ICUs.

The most common sources of infection were pneumonia (47.9%) and urinary tract infection (37.5%). In addition, patients frequently presented with acute clinical conditions associated with ICU admission, including acute decompensated heart failure (29.2%), acute kidney injury (20.8%), COPD exacerbation (18.8%), respiratory failure (18.8%), and hyperglycemic crises (12.5%). The majority of patients had multimorbidity, with the most common comorbidities including diabetes mellitus, hypertension, hyperlipidemia, and coronary artery disease. The demographic and clinical characteristics of the patients are presented in Table 1, including primary sources of infection, acute clinical conditions associated with ICU admission, and underlying comorbidities.

Table 2 presents a comparison of laboratory parameters obtained at the initial (0 h) intensive care unit admission and at the 48th hour of hospitalization.

### 3.2. Hematological Parameters

At 48 h, hemoglobin, hematocrit, and platelet levels showed a significant decrease compared to baseline values (*p* < 0.05). No statistically significant changes were observed in white blood cell, neutrophil, or lymphocyte counts (*p* > 0.05), as shown in Table 2.

### 3.3. Biochemical and Inflammatory Markers and Relaxin-1 Levels

Significant reductions were observed in urea, calcium, total bilirubin, LDH, CRP, and INR levels at 48 h compared to baseline (*p* < 0.05). In contrast, liver enzymes, creatinine, albumin, glucose, procalcitonin, and NT-proBNP levels did not change significantly over time (*p* > 0.05), as shown in Table 2.

**Figure 1:**
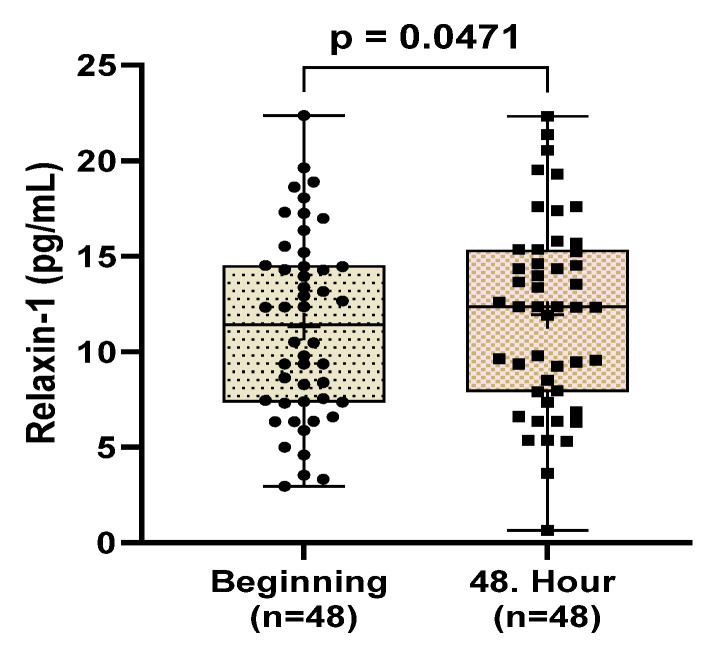
Changes in serum Relaxin-1 levels between admission and 48 h in patients with sepsis and septic shock.

Relaxin-1 levels increased significantly from baseline to 48 h (*p* = 0.047) (Figure 1). Similarly, SOFA and APACHE II scores showed significant reductions over the same period (*p* < 0.001). Overall, these findings demonstrate dynamic changes in hematological, biochemical, and inflammatory parameters during the early course of sepsis and septic shock.

### 3.4. Comparison Between Survivors and Non-Survivors

Patients were categorized as survivors (*n* = 32) and non-survivors (*n* = 16) based on ICU mortality. The mean age was higher in non-survivors compared to survivors (78.44 ± 12.12 vs. 70.81 ± 15.36 years), although this difference did not reach statistical significance (*p* = 0.069). The length of ICU stay was significantly longer in non-survivors compared to survivors (4.38 ± 7.21 days) (*p* = 0.021). Comparison between groups revealed significant differences in several hematological, biochemical, and clinical parameters. Non-survivors had significantly lower hemoglobin, hematocrit, and albumin levels, whereas platelet count, total bilirubin, LDH, CRP, INR, troponin, and Relaxin-1 levels were significantly higher compared to survivors (*p* < 0.05). Additionally, SOFA and APACHE II scores were significantly higher in non-survivors compared to survivors (*p* < 0.001). Detailed comparisons of laboratory and clinical parameters between the two groups are presented in Table 3.

**Figure 2:**
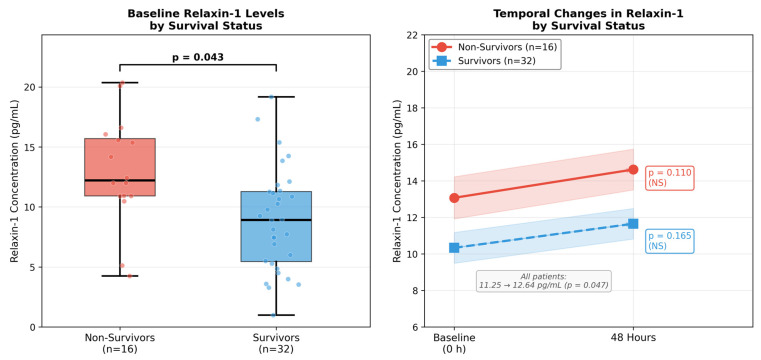
Comparison of baseline and temporal changes in serum Relaxin-1 levels according to survival status. Baseline Relaxin-1 levels were significantly higher in non-survivors compared to survivors (*p* = 0.043). Changes in Relaxin-1 levels from admission to 48 h did not differ significantly within groups (survivors: *p* = 0.165; non-survivors: *p* = 0.110). Data are presented with standard error bars.

Relaxin-1 levels were significantly higher in non-survivors compared to survivors (14.62 ± 4.47 pg/mL vs. 11.65 ± 4.73 pg/mL, *p* = 0.043) (Figure 2). In addition, disease severity scores were markedly elevated in non-survivors. The mean SOFA score was significantly higher in non-survivors than in survivors (13.94 ± 1.77 vs. 8.15 ± 3.30, *p* < 0.001). Similarly, the mean APACHE II score was significantly higher in non-survivors (29.38 ± 9.17) compared to survivors (19.31 ± 8.24) (*p* < 0.001).

Higher Relaxin-1 levels were observed in patients with increased disease severity and ICU mortality.

### 3.5. Predictive Value of Relaxin-1 for ICU Mortality: ROC Curve Analysis

**Figure 3:**
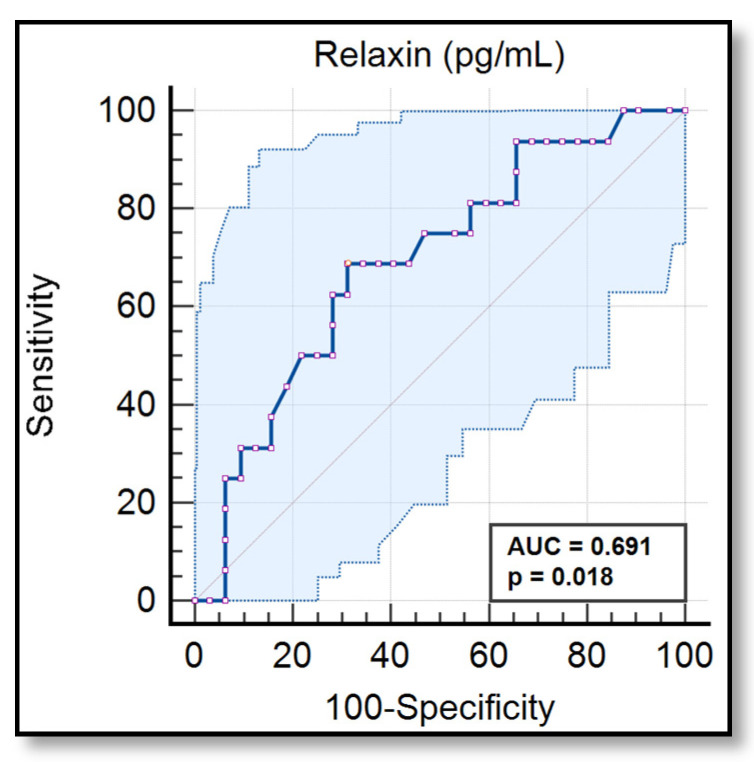
Receiver operating characteristic curve analysis of serum Relaxin-1 levels for predicting ICU mortality. The solid blue line represents the receiver operating characteristic (ROC) curve, the shaded area indicates the 95% confidence interval, and the diagonal gray line represents the reference line.

Receiver operating characteristic (ROC) curve analysis was performed to evaluate the ability of Relaxin-1 levels to discriminate between survivors and non-survivors (Figure 3). The area under the curve (AUC) was 0.691 (95% CI: 0.542–0.817; *p* = 0.018), indicating a moderate discriminative performance.

The optimal cutoff value determined by the Youden index was >13.64 pg/mL, yielding a sensitivity of 68.75% and a specificity of 68.75%. The positive likelihood ratio was 2.20, and the negative likelihood ratio was 0.45.

These findings suggest that Relaxin-1 has limited but statistically significant ability to discriminate ICU mortality. However, given its moderate sensitivity and specificity, Relaxin-1 alone may not be sufficient as a standalone prognostic marker and should be interpreted in conjunction with established clinical scoring systems.

To further evaluate the prognostic contribution of Relaxin-1 independent of established severity markers, binary logistic regression was performed with ICU mortality as the dependent variable. Given the limited number of outcome events (*n* = 16), the model was restricted to two covariates. In the adjusted model including APACHE II score and serum albumin, APACHE II was a significant independent predictor of mortality (OR = 1.14, 95% CI: 1.05–1.23; *p* = 0.002), as was serum albumin (OR = 0.86, 95% CI: 0.80–0.93; *p* < 0.001). The formal independent assessment of Relaxin-1 within a multivariable model was precluded by the insufficient events-per-variable ratio (16 events/3 predictors = 5.3, below the recommended minimum of 10). These results must therefore be considered exploratory and hypothesis-generating.

**Figure 4:**
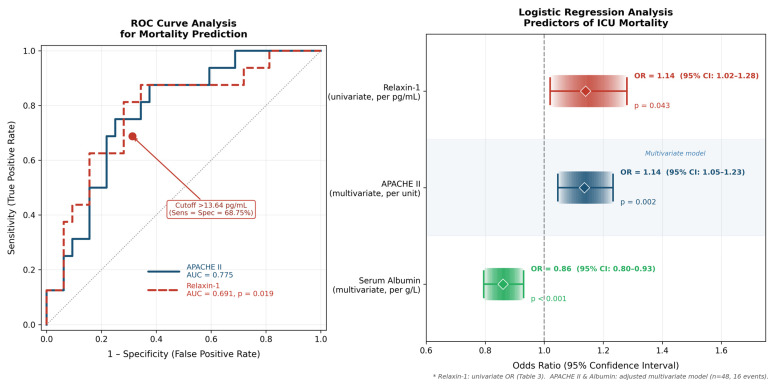
(**Left**) ROC curve analysis comparing the discriminative ability of Relaxin-1 (AUC = 0.691, *p* = 0.019) and APACHE II score (AUC = 0.791) for predicting ICU mortality. The blue solid line represents the APACHE II ROC curve, the red dashed line represents the Relaxin–1 ROC curve, the diagonal gray line indicates the reference line, and the red dot indicates the optimal Relaxin-1 cutoff (>13.64 pg/mL). (**Right**) Forest plot of odds ratios from univariate Relaxin-1 and multivariate logistic regression (APACHE II, serum albumin). The colored boxes and horizontal bars represent odds ratios and 95% confidence intervals for each predictor. * Relaxin–1 OR derived from univariate analysis; APACHE II and albumin ORs from the adjusted multivariate model.

For comparison, ROC curve analysis demonstrated that APACHE II had an AUC of 0.791 for predicting ICU mortality, compared with an AUC of 0.691 for Relaxin-1. Although the AUC for APACHE II was numerically higher, a formal statistical comparison of the two AUCs was not performed given the exploratory nature of the study and the small sample size. These findings suggest that Relaxin-1, while statistically associated with mortality in univariate analysis, likely reflects overall disease severity rather than providing independent prognostic information beyond established severity scores. The results are visualized in Figure 4.

**Figure 5:**
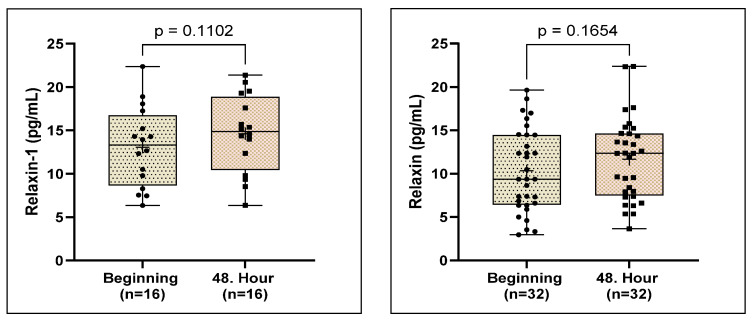
Temporal changes in serum Relaxin-1 levels from baseline to 48 h in survivors and non-survivors. No significant within-group differences were observed.

Changes in Relaxin1 levels over time were analyzed separately in survivors and non-survivors. In non-survivors, mean Relaxin-1 levels increased from 13.07 ± 4.61 pg/mL at baseline to 14.62 ± 4.47 pg/mL at 48 h; however, this change was not statistically significant (*p* = 0.110). Similarly, in survivors, Relaxin-1 levels increased from 10.33 ± 4.77 pg/mL to 11.65 ± 4.73 pg/mL, without reaching statistical significance (*p* = 0.165). These findings indicate that no significant within-group change in Relaxin-1 levels occurred over the 48 h period (Figure 5).

## 4. Discussion

To the best of our knowledge, this is among the first clinical studies to report an association between circulating Relaxin-1 levels and hemodynamic and inflammatory alterations in patients with sepsis and septic shock. Early recognition of sepsis and a deeper understanding of its pathophysiological mechanisms remain essential to reduce morbidity and mortality in this high-risk population.

The progression from sepsis to septic shock is characterized by profound alterations in vasoactive inflammatory mediators, resulting in cellular dysfunction and metabolic derangements [12]. Dysregulated activation of chemokines plays a central role in this process; macrophage-derived MIP-2 contributes to neutrophil recruitment via CXCR1 and CXCR2 receptors and is associated with organ damage and mortality [13]. Although biomarkers such as pro- and anti-inflammatory cytokines, C-reactive protein (CRP), and procalcitonin (PCT) are routinely used in clinical practice, the complex and heterogeneous nature of septic shock highlights the need for novel biomarkers that reflect both inflammatory and hemodynamic disturbances [14].

Relaxin-1 is a pleiotropic peptide hormone with anti-inflammatory, antifibrotic, angiogenic, vasodilatory, and cytoprotective properties [15,16]. Its biological actions are primarily mediated through binding to the G protein-coupled receptor RXFP1, which activates multiple intracellular signaling pathways, including cAMP, cGMP, and MAPK cascades. Activation of these pathways modulates gene expression involved in extracellular matrix turnover, vascular remodeling, and endothelial homeostasis, including regulation of TGF-β signaling, matrix metalloproteinases, angiogenic growth factors, and endothelin receptor expression [16]. Through these mechanisms, relaxin contributes to the preservation of vascular integrity and endothelial function under inflammatory stress conditions. Given that endothelial dysfunction and microvascular dysregulation are central determinants of organ failure and mortality in septic shock, these biological properties provide a plausible mechanistic link between Relaxin-1 and disease severity in septic shock.

Experimental studies further demonstrate that relaxin reduces leukocyte migration—particularly neutrophil infiltration—via nitric oxide (NO)-dependent mechanisms [17] and modulates macrophage secretion of pro-inflammatory cytokines [18]. Under inflammatory stress conditions, relaxin attenuates endothelial dysfunction by decreasing endothelin-1 expression, oxidative stress markers, and arginase II activity, thereby improving NO bioavailability [19]. In cardiovascular settings, especially in heart failure models, relaxin has been shown to induce systemic and coronary vasodilation, enhance cardiac output, and improve vascular compliance through nitric oxide-mediated pathways [20]. It has also been reported to reduce pulmonary artery pressure and improve hemodynamics [21], enhance splanchnic blood flow and glucocorticoid receptor-mediated anti-inflammatory responses, and attenuate vascular inflammation by suppressing adhesion molecules such as VCAM-1 and chemokines such as MCP-1 [22]. Additionally, protective effects against microvascular injury and ischemia–reperfusion damage have been demonstrated in cardiac and renal experimental models [10,23,24].

In the present study, Relaxin-1 levels were significantly higher in non-survivors compared to survivors, and concentrations demonstrated an increasing trend during the first 48 h in both groups. While this association is statistically significant in univariate analysis, it is important to interpret this finding with caution. Given the significantly higher APACHE II and SOFA scores observed in the non-survivor group, elevated Relaxin-1 levels likely reflect the overall magnitude of critical illness severity rather than functioning as an independent driver of mortality. These findings suggest that circulating Relaxin-1 levels may reflect ongoing hemodynamic and inflammatory alterations during the early course of sepsis and septic shock.

Interestingly, despite its predominantly protective vasodilatory and anti-inflammatory properties, elevated Relaxin-1 levels were associated with mortality.

This observation may reflect the magnitude of systemic inflammatory and hemodynamic stress. Therefore, increased Relaxin-1 concentrations in non-survivors may reflect greater disease severity rather than a deleterious effect of the hormone itself. Furthermore, the observed increasing trend of Relaxin-1 levels within the first 48 h aligns with the period of maximal hemodynamic instability in many patients. However, as our study did not employ a formal longitudinal framework—such as mixed-effects modeling—to statistically compare these trajectories between groups, these temporal findings should be interpreted as descriptive observations and require confirmation in larger studies.

Taken together, our findings suggest that Relaxin-1 may be associated with disease severity in sepsis and septic shock, potentially reflecting the interplay between inflammatory activation and vascular dysregulation. However, our results should be interpreted as hypothesis-generating; while Relaxin-1 is associated with adverse outcomes in this cohort, its potential incremental prognostic value—beyond established scores like APACHE II—could not be established in the present study. Larger multicenter studies are warranted to clarify its independent prognostic significance and to determine whether relaxin represents primarily a marker of disease severity or a mediator within the pathophysiological cascade.

In the present study, multivariate logistic regression confirmed that APACHE II score independently predicted mortality after adjustment for serum albumin (OR = 1.14, 95% CI: 1.05–1.23; *p* = 0.002). Although Relaxin-1 was significantly higher in non-survivors on univariate analysis (*p* = 0.043), its independent contribution in a multivariable framework could not be formally established due to the limited number of outcome events relative to the number of candidate predictors (events-per-variable ratio: 16/3 = 5.3, below the widely recommended minimum of 10). This is a recognized limitation of small, exploratory studies and does not preclude the biological plausibility of Relaxin-1 as a prognostic marker. Future studies should focus on the serial measurement of Relaxin-1 in conjunction with advanced bedside hemodynamic monitoring and formal evaluation of added discrimination indices to better define its role as a potential marker of disease severity.

## 5. Limitations

Several limitations of the present study should be considered.

First, the sample size was small (*n* = 48), with only 16 mortality events. This restricted the complexity of multivariable models and precluded definitive conclusions regarding the independent prognostic value of Relaxin-1 after adjustment for established severity indicators such as SOFA and APACHE II scores. Future studies should be adequately powered, with a pre-specified sample size calculation enabling robust multivariable analyses with at least 10 events per predictor variable.

Second, this was a single-center study conducted in a Level II intensive care unit, which may limit the generalizability of findings. A multicenter design with broader patient enrollment would strengthen the external validity of these observations.

Third, treatment-related confounders were not systematically recorded in the analysis. Specifically, vasopressor doses, fluid balance, detailed antimicrobial regimens, corticosteroid use, mechanical ventilation parameters, and renal replacement therapy protocols are recognized determinants of outcome in septic shock and may influence Relaxin-1 levels. Future studies should collect and report these variables to enable meaningful multivariable adjustment.

Fourth, although Relaxin-1 was measured at two time points (admission and 48 h), a formal repeated-measures analysis incorporating a group-by-time interaction term was not performed, as the sample size was insufficient to power such an analysis. The temporal trajectory of Relaxin-1 within each survival group therefore requires evaluation in larger longitudinal studies with more frequent sampling and appropriate mixed-effects modeling.

Fifth, while patients receiving chemotherapy or radiotherapy were excluded, corticosteroids were administered to some patients as part of standard septic shock management. The potential confounding effect of corticosteroid use on Relaxin-1 levels and immune responses was not specifically evaluated in this study. Future research should stratify analyses by corticosteroid use to clarify this potential interaction.

Sixth, a discrepancy in septic shock classification was noted, with six patients (12.5%) in the cohort not meeting strict Sepsis-3 criteria for septic shock at the time of data extraction (Table 1: Septic Shock: Yes *n* = 42, No *n* = 6). These patients met sepsis criteria but not the vasopressor and lactate thresholds defining septic shock. This introduces a degree of clinical heterogeneity and may affect the generalizability of Relaxin-1 findings specifically to the septic shock population. Future studies should apply and document inclusion criteria prospectively and rigorously at the point of enrollment.

Seventh, Relaxin-1 measurements were performed in a single run without duplicate testing. While this is consistent with the exploratory nature of the study, it represents an analytical limitation, as intra-assay variability could not be formally assessed. Future studies employing high-sensitivity assays should consider duplicate or triplicate testing to ensure maximal analytical precision.

## 6. Conclusions

Sepsis and septic shock are characterized by complex interactions between inflammatory mediators and hemodynamic dysregulation, with marked heterogeneity in the host response. Although current management strategies focus on infection source control, appropriate antimicrobial therapy, and hemodynamic stabilization, improving early risk stratification and understanding the underlying pathophysiological mechanisms remain critical challenges.

In this prospective study, circulating Relaxin-1 levels were significantly higher in non-survivors among patients with sepsis and septic shock, demonstrating an association with mortality in univariate analyses. Given their established roles in inflammation, endothelial function, and vascular regulation, elevated Relaxin-1 levels likely reflect the overall severity of inflammatory and hemodynamic disturbances rather than functioning as an independent prognostic driver.

While the APACHE II score remains a robust independent predictor of mortality, the dynamic profile of Relaxin-1 offers a biologically plausible link to endothelial dysfunction and disease severity. These findings suggest that Relaxin-1 may represent a biologically relevant marker associated with adverse outcomes and may have potential value in monitoring disease progression as a surrogate of host stress response. However, it is important to emphasize that this study does not establish Relaxin-1 as an independent prognostic biomarker beyond established clinical scores.

Larger and more comprehensive studies are required to confirm its potential incremental prognostic significance and to further elucidate its role in the pathophysiology of sepsis and septic shock. Specifically, future research should employ larger cohorts and formal modeling to determine whether Relaxin-1 provides added discriminative value beyond traditional severity indicators.

## Figures and Tables

**Table 1:** Demographic and Clinical Characteristics of the Patients (*n* = 48).

Variable	Category	Number (*n*)	Percentage (%)
**Sex**	Female	26	54.2
	Male	22	45.8
**Reason for ICU Admission**			
**Primary infectious diagnoses**	Pneumonia	23	47.9
	Urinary tract infection	18	37.5
	Other infections	7	14.6
**Acute clinical conditions associated with ICU admission**	AKI	10	20.8
	Acute Decompensated Heart Failure	14	29.2
	COPD exacerbation	9	18.8
	Respiratory failure	9	18.8
	Hyperglycemic crises	6	12.5
Comorbidities *	DM + HT	36	75.0
	DM + HT + CAD	29	60.4
	DM + HT + CKD	5	10.4
	DM + HT + Congestive Heart Failure	18	37.5
	COPD	4	8.3
	Dementia	6	12.5
	CKD	10	20.8
	Cirrhosis	1	2.1
	Rheumatologic diseases	4	8.3
**Culture Positivity**	Present	38	79.2
	Absent	10	20.8
**Septic Shock**	Yes	42	87.5
	No	6	12.5
**Mechanical Ventilation**	Yes	18	37.5
	No	30	62.5
**Hemodialysis**	Yes	8	16.7
	No	40	83.3
**ICU Mortality Outcome**	Survivors	32	66.7
	Non-survivors	16	33.3

* Comorbidities are presented as recorded clinical combinations; therefore, categories are not mutually exclusive, and some patients may be included in more than one group. DM, diabetes mellitus; HT, hypertension; CAD, coronary artery disease; CKD, chronic kidney disease; COPD, chronic obstructive pulmonary disease; AKI, acute kidney injury; ICU, intensive care unit.

**Table 2:** Comparison of baseline and 48 h laboratory parameters in patients with sepsis and septic shock.

Variable	Sepsis Status		*p* Value
	At Admission (*n* = 48)	48 h (*n* = 48)	
	Mean ± SD|Median (Min–Max) IQR		
WBC (×10^3^/µL)	12.10 ± 5.54	11.51 ± 6.00	0.601
Hb (g/dL)	11.47 ± 2.56	10.98 ± 1.86	0.003 *
Hct (%)	34.94 ± 7.28	32.57 ± 5.42	0.002 *
PLT (×10^3^/µL)	297.63 ± 84.03	226.53 ± 112.44	0.001 *
NEU (×10^3^/µL)	10.01 ± 5.19	9.69 ± 5.48	0.776
LYM (×10^3^/µL)	1.10 (0.20–3.00) [0.60–1.60]	0.90 (0.20–2.80) [0.50–1.40]	0.188 ^#^
PDW (%)	11.24 ± 1.08	13.29 ± 3.70	0.001 *
Glucose (mg/dL)	180 (55–428) [95–225]	165 (60–400) [100–210]	0.484 ^#^
ALT (U/L)	38.23 ± 9.24	40.63 ± 29.19	0.101
AST (U/L)	37.71 ± 12.13	38.84 ± 20.63	0.724
Urea (mg/dL)	65 (21–239) [40–95]	45 (15–180) [30–80]	0.002 *^,#^
Creatinine (mg/dL)	1.80 (0.60–12.0) [1.10–2.80]	1.40 (0.50–6.50) [0.90–2.10]	0.101^#^
Albumin (g/dL)	31.56 ± 5.19	29.87 ± 6.30	0.143
ALP (U/L)	112.25 ± 27.86	118.78 ± 164.43	0.774
GGT (U/L)	95.38 ± 53.14	86.25 ± 130.56	0.628
Ca (mg/dL)	9.52 ± 0.53	8.25 ± 0.84	<0.001 *
Total Bilirubin (mg/dL)	1.28 ± 0.23	0.76 ± 0.67	<0.001 *
LDH (U/L)	283.31 ± 73.45	209.00 ± 78.59	<0.001 *
CRP (mg/L)	85 (5–360) [30–180]	65 (4–290) [20–140]	0.003 *^,#^
PCT (ng/mL)	2.50 (0.10–96) [0.90–6.20]	1.80 (0.10–18) [0.80–5.90]	0.791 ^#^
NT-proBNP (pg/mL)	4500 (35–38,908) [1200–12,000]	4800 (40–35,000) [1300–11,000]	0.673 ^#^
INR	1.60 ± 0.55	1.15 ± 0.68	<0.001 *
Relaxin-1 (pg/mL)	11.25 ± 4.85	12.64 ± 4.81	0.047 *
SOFA score	12.75 ± 3.35	8.80 ± 3.95	<0.001 *
APACHE-II score	24.97 ± 10.13	18.06 ± 7.12	<0.001 *

* The Paired *t* test and ^#^ Wilcoxon Sign Rank test were applied, and a *p*-value < 0.05 was considered statistically significant. WBC, white blood cell count; Hb, hemoglobin; Hct, hematocrit; PLT, platelet count; NEU, neutrophil count; LYM, lymphocyte count; PDW, platelet distribution width; ALT, alanine aminotransferase; AST, aspartate aminotransferase; ALP, alkaline phosphatase; GGT, gamma-glutamyl transferase; Ca, calcium; LDH, lactate dehydrogenase; CRP, C-reactive protein; PCT, procalcitonin; NT-proBNP, N-terminal pro–B-type natriuretic peptide; INR, international normalized ratio; SOFA, Sequential Organ Failure Assessment; APACHE II, Acute Physiology and Chronic Health Evaluation II.

**Table 3:** Comparison of baseline clinical and laboratory parameters between survivors and non-survivors.

Variable	Survival Status		*p* Value
	Non-Survivors (*n* = 16)	Survivors (*n* = 32)	
	Mean ± SD|Median (Min–Max) [IQR]		
Age (years)	78.44 ± 12.12	70.81 ± 15.36	0.069
Length of ICU stay (day)	6.50 (1–45) [2.25–17.50]	2.00 (1–40) [1.00–4.75]	0.021 *^,#^
WBC (×10^3^/µL)	15.55 ± 6.15	12.29 ± 5.71	0.062
Hb (g/dL)	9.37 ± 1.00	11.03 ± 2.44	0.002 *
Hct (%)	28.61 ± 3.28	33.73 ± 6.88	0.001 *
PLT (×10^3^/µL)	356.63 ± 142.32	247.03 ± 89.24	0.008 *
NEU (×10^3^/µL)	13.77 ± 5.17	10.31 ± 5.50	0.032 *
LYM (×10^3^/µL)	0.80 (0.20–2.60) [0.30–1.45]	1.10 (0.20–3.00) [0.60–1.60]	0.335 ^#^
Glucose (mg/dL)	149.50 (81–360) [116.75–224.00]	119.50 (55–428) [91.25–152.75]	0.092 ^#^
ALT (U/L)	44.20 ± 36.87	39.16 ± 16.63	0.553
AST (U/L)	54.78 ± 19.63	45.30 ± 18.78	0.093
Urea (mg/dL)	91.50 (34–184) [58.75–127.75]	62.50 (21–239) [35.25–84.75]	0.167 ^#^
Creatinine (mg/dL)	2.13 ± 1.36	1.55 ± 1.14	0.160
Albumin (g/dL)	27.58 ± 4.40	31.86 ± 6.17	0.008 *
Total Bilirubin (mg/dL)	1.84 ± 0.83	0.88 ± 0.38	<0.001 *
LDH (U/L)	344.13 ± 87.32	282.69 ± 68.13	0.012 *
CRP (mg/L)	150.00 (25–293) [94.00–202.50]	71.50 (5–360) [20.00–132.50]	0.047 *^,#^
PCT (ng/mL)	1.79 (0.10–96) [0.70–6.32]	2.75 (0.10–18) [1.07–6.17]	0.318 ^#^
NT-proBNP (pg/mL)	5216.00 (534–34,468) [1823.00–12,913.25]	3753.00 (35–38,908) [1030.75–10,016.00]	0.534 ^#^
Uric Acid (mg/dL)	7.20 ± 2.54	6.56 ± 2.35	0.385
Vit B12 (pg/mL)	618.50 (264–1869) [428.25–1114.50]	458.50 (197–1316) [355.75–600.25]	0.045 *^,#^
TSH (µIU/mL)	1.22 (0.28–4.12) [0.63–2.20]	1.40 (0.05–12.20) [0.79–3.01]	0.119 ^#^
Ferritin (ng/mL)	324.00 (57–1421) [182.50–654.25]	178.50 (32–1226) [106.25–354.75]	0.110 ^#^
INR	1.77 ± 0.82	1.30 ± 0.49	0.049 *
Troponin (ng/L)	32.50 (4–1116) [11.25–126.75]	20.00 (3–130) [14.00–43.50]	0.005 *^,#^
Relaxin-1 (pg/mL)	14.62 ± 4.47	11.65 ± 4.73	0.043 *
SOFA score	13.94 ± 1.77	8.15 ± 3.30	<0.001 *
APACHE-II score	29.38 ± 9.17	19.31 ± 8.24	<0.001 *

* *p* < 0.05 was considered statistically significant. The independent *t* test and ^#^ Mann–Whitney U test were applied, and a *p*-value < 0.05 was considered statistically significant. ICU, intensive care unit; WBC, white blood cell count; Hb, hemoglobin; Hct, hematocrit; PLT, platelet count; NEU, neutrophil count; LYM, lymphocyte count; ALT, alanine aminotransferase; AST, aspartate aminotransferase; LDH, lactate dehydrogenase; CRP, C-reactive protein; PCT, procalcitonin; NT-proBNP, N-terminal pro–B-type natriuretic peptide; Vit B12, vitamin B12; TSH, thyroid-stimulating hormone; INR, international normalized ratio; SOFA, Sequential Organ Failure Assessment; APACHE II, Acute Physiology and Chronic Health Evaluation II.

## Data Availability

The datasets generated and/or analyzed during the current study are available from the corresponding author upon reasonable request.

## Abbreviations

The following abbreviations are used in this manuscript:

CRP	C-reactive protein
PCT	Procalcitonin
MIP-2	Macrophage Inflammatory Protein-2
CXCR1	C-X-C Motif Chemokine Receptor 1
CXCR2	C-X-C Motif Chemokine Receptor 2
NO	Nitric Oxide
TNF-α	Tumor Necrosis Factor-alpha
SOFA	Sequential Organ Failure Assessment
APACHE II	Acute Physiology and Chronic Health Evaluation II
RXFP1	Relaxin Family Peptide Receptor 1
cAMP	Cyclic Adenosine Monophosphate
cGMP	Cyclic Guanosine Monophosphate
MAPKs	Mitogen-Activated Protein Kinases
TGF-β	Transforming Growth Factor-beta
MMPs	Matrix Metalloproteinases
VCAM-1	Vascular Cell Adhesion Molecule-1
MCP-1	Monocyte Chemoattractant Protein-1
VEGF	Vascular Endothelial Growth Factor

## References

[1] Chiscano-Camón L., Plata-Menchaca E., Ruiz-Rodríguez J.C., Ferrer R. (2022). Fisiopatología del shock séptico [Pathophysiology of septic shock]. Med. Intensiv. (Engl. Ed.).

[2] Wiersinga W.J., van der Poll T. (2022). Immunopathophysiology of human sepsis. eBioMedicine.

[3] Font M.D., Thyagarajan B., Khanna A.K. (2020). Sepsis and Septic Shock—Basics of diagnosis, pathophysiology and clinical decision making. Med. Clin. North Am..

[4] Jarczak D., Kluge S., Nierhaus A. (2021). Sepsis-Pathophysiology and Therapeutic Concepts. Front. Med..

[5] Pandey S., Adnan Siddiqui M., Azim A., Trigun S.K., Sinha N. (2021). Serum metabolic profiles of septic shock patients based upon co-morbidities and other underlying conditions. Mol. Omics.

[6] Chen T.Y., Li X., Hung C.H., Bahudhanapati H., Tan J., Kass D.J., Zhang Y. (2020). The relaxin family peptide receptor 1 (RXFP1): An emerging player in human health and disease. Mol. Genet. Genom. Med..

[7] Patil N.A., Rosengren K.J., Separovic F., Wade J.D., Bathgate R.A.D., Hossain M.A. (2017). Relaxin family peptides: Structure-activity relationship studies. Br. J. Pharmacol..

[8] Sankaran S. (2012). Creasy and Resnik’s Maternal–Fetal Medicine: Principles and Practice Sixth edition. Obstet. Med..

[9] Goldsmith L.T., Weiss G. (2009). Relaxin in human pregnancy. Ann. N. Y. Acad. Sci..

[10] Abdel-Magid A.F. (2025). The Relaxin Family Peptide Receptor 1 (RXFP1) Agonists as Potential Treatment for Heart Failure. ACS Med. Chem. Lett..

[11] Bakker J., Kattan E., Annane D., Castro R., Cecconi M., De Backer D., Dubin A., Evans L., Gong M.N., Hamzaoui O. (2022). Current practice and evolving concepts in septic shock resuscitation. Intensive Care Med..

[12] Russell J.A., Rush B., Boyd J. (2018). Pathophysiology of Septic Shock. Crit. Care Clin..

[13] Joffre J., Hellman J., Ince C., Ait-Oufella H. (2020). Endothelial Responses in Sepsis. Am. J. Respir. Crit. Care Med..

[14] Gorecki G., Cochior D., Moldovan C., Rusu E. (2021). Molecular mechanisms in septic shock (Review). Exp. Ther. Med..

[15] Martin B., Gabris-Weber B.A., Reddy R., Romero G., Chattopadhyay A., Salama G. (2018). Relaxin reverses inflammatory and immune signals in aged hearts. PLoS ONE.

[16] Sarwar M., Du X.J., Dschietzig T.B., Summers R.J. (2017). The actions of relaxin on the human cardiovascular system. Br. J. Pharmacol..

[17] Masini E., Nistri S., Vannacci A., Bani Sacchi T., Novelli A., Bani D. (2004). Relaxin inhibits the activation of human neutrophils: Involvement of the nitric oxide pathway. Endocrinology.

[18] Horton J.S., Yamamoto S.Y., Bryant-Greenwood G.D. (2011). Relaxin modulates proinflammatory cytokine secretion from human decidual macrophages. Biol. Reprod..

[19] Dschietzig T., Brecht A., Bartsch C., Baumann G., Stangl K., Alexiou K. (2012). Relaxin improves TNF-α-induced endothelial dysfunction: The role of glucocorticoid receptor and phosphatidylinositol 3-kinase signalling. Cardiovasc. Res..

[20] Teichman S.L., Unemori E., Dschietzig T., Conrad K., Voors A.A., Teerlink J.R., Felker G.M., Metra M., Cotter G. (2009). Relaxin, a pleiotropic vasodilator for the treatment of heart failure. Heart Fail Rev..

[21] Eachempati S.R., Hydo L., Barie P.S. (1999). Gender-based differences in outcome in patients with sepsis. Arch. Surg..

[22] Brecht A., Bartsch C., Baumann G., Stangl K., Dschietzig T. (2011). Relaxin inhibits early steps in vascular inflammation. Regul. Pept..

[23] Gao X.M., Su Y., Moore S., Han L.-P., Kiriazis H., Lu Q., Zhao W.-B., Ruze A., Fang B.-B., Duan M.-J. (2019). Relaxin mitigates microvascular damage and inflammation following cardiac ischemia-reperfusion. Basic Res. Cardiol..

[24] Ghosh R.K., Banerjee K., Tummala R., Ball S., Ravakhah K., Gupta A. (2017). Serelaxin in acute heart failure: Most recent update on clinical and preclinical evidence. Cardiovasc Ther..

